# Chimeras of GROWTH-REGULATING FACTOR and GRF-INTERACTING FACTOR enhance leaf-based regeneration and transformation efficiency in tomato

**DOI:** 10.1093/jxb/erag187

**Published:** 2026-04-22

**Authors:** Sanghee Lee, Ah-Hyeon Choi, Minah Jung, Hualin Nie, Seo-Rin Ko, Nayoung Kim, Suk-Yoon Kwon, Ah-Young Shin

**Affiliations:** Plant Systems Engineering Research Center, Korea Research Institute of Bioscience and Biotechnology (KRIBB), Daejeon 34141, Republic of Korea; Department of Functional Genomics, KRIBB School of Bioscience, University of Science and Technology (UST), 217 Gajeong-ro Yuseong-gu, Daejeon 34113, Republic of Korea; Plant Systems Engineering Research Center, Korea Research Institute of Bioscience and Biotechnology (KRIBB), Daejeon 34141, Republic of Korea; Department of Functional Genomics, KRIBB School of Bioscience, University of Science and Technology (UST), 217 Gajeong-ro Yuseong-gu, Daejeon 34113, Republic of Korea; Plant Systems Engineering Research Center, Korea Research Institute of Bioscience and Biotechnology (KRIBB), Daejeon 34141, Republic of Korea; Plant Systems Engineering Research Center, Korea Research Institute of Bioscience and Biotechnology (KRIBB), Daejeon 34141, Republic of Korea; Plant Systems Engineering Research Center, Korea Research Institute of Bioscience and Biotechnology (KRIBB), Daejeon 34141, Republic of Korea; Department of Functional Genomics, KRIBB School of Bioscience, University of Science and Technology (UST), 217 Gajeong-ro Yuseong-gu, Daejeon 34113, Republic of Korea; Plant Systems Engineering Research Center, Korea Research Institute of Bioscience and Biotechnology (KRIBB), Daejeon 34141, Republic of Korea; Plant Systems Engineering Research Center, Korea Research Institute of Bioscience and Biotechnology (KRIBB), Daejeon 34141, Republic of Korea; Department of Biological Sciences, Sungkyunkwan University, 2066 Seobu-ro, Suwon 16419, Republic of Korea; Plant Systems Engineering Research Center, Korea Research Institute of Bioscience and Biotechnology (KRIBB), Daejeon 34141, Republic of Korea; Department of Functional Genomics, KRIBB School of Bioscience, University of Science and Technology (UST), 217 Gajeong-ro Yuseong-gu, Daejeon 34113, Republic of Korea; Department of Biological Sciences, Sungkyunkwan University, 2066 Seobu-ro, Suwon 16419, Republic of Korea; Cardiff University, UK

**Keywords:** GRF–GIF chimera, RNA stability, tomato regeneration, transcriptome profiling, ubiquitin–proteasome system

## Abstract

Efficient regeneration remains a major constraint for tomato (*Solanum lycopersicum*) transformation, particularly from leaf explants. Here, we evaluated chimeric GROWTH-REGULATING FACTOR (GRF)–GRF-INTERACTING FACTOR (GIF) proteins as regeneration enhancers and examined regulatory features underlying their activity. Fusion constructs based on *Arabidopsis thaliana GRF5–GIF1* and their tomato homologs *SlGRF4* and *SlGIF1b* markedly increased shoot regeneration from leaf explants, with similar enhancement observed in cotyledon-derived tissues, across three tomato cultivars. Regenerated shoots were able to form roots, indicating that GRF–GIF-mediated regeneration produced developmentally competent plantlets. Temporal expression profiling indicated that GRF–GIF transcript levels increased during regeneration and coincided with the induction of cytokinin- and auxin-associated regulators linked to meristem initiation. RNA-seq analyses revealed a shared early transcriptional program enriched for transcription factor activity and protein homeostasis, accompanied by broad repression of metabolism-related pathways. Despite strong phenotypic effects, GRF–GIF fusion proteins accumulated poorly in stable transformants. Transient expression assays suggested that their abundance is influenced by the ubiquitin–proteasome system, and mutational analyses identified lysine residues affecting protein stability, although stabilization did not further enhance regeneration. Together, these findings support the use of GRF–GIF chimeras as effective enhancers of tomato regeneration from leaf explants and highlight the contribution of coordinated regulation at the transcript and protein levels to their functional window during cellular reprogramming.

## Teaser

GRF–GIF chimeras enhance shoot regeneration from tomato leaf explants, and this regeneration is consistent across multiple cultivars. Transcript and protein stability are closely associated with the GRF–GIF functional window.

## Introduction

Efficient plant regeneration is a fundamental requirement for genetic transformation, genome editing, and other biotechnological applications in crops. However, although tomato (*Solanum lycopersicum*) is generally amenable to genetic transformation, regeneration efficiency remains highly genotype and explant dependent, leading to substantial variability across cultivars and experimental systems. Cotyledon and hypocotyl explants have historically been used because of their relatively high responsiveness to shoot induction protocols (Sun *et al.*, 2006). In contrast, true leaf explants can be obtained in greater numbers per seedling and are less strictly constrained by developmental stage, but they generally exhibit lower regenerative competence and unstable shoot conversion compared with cotyledon or hypocotyl explants, making them a key target for regeneration improvement strategies (Sandhya *et al.*, 2022). As a result, improving regeneration from leaf explants is considered important for increasing throughput and flexibility in tomato transformation pipelines.

Manipulation of transcriptional regulators involved in cell fate reprogramming and organogenesis has been proposed as a promising approach to overcome these limitations. Members of the GROWTH-REGULATING FACTOR (GRF) family and their co-activators, GRF-INTERACTING FACTORs (GIFs), have been identified as critical determinants of plant growth and development (Kim *et al.*, 2003; Lee *et al.*, 2009; Omidbakhshfard *et al.*, 2015). GRFs function as transcription factors that control cell proliferation and differentiation, whereas GIFs act as co-activators that enhance GRF activity. In several species, including Arabidopsis, lettuce, and watermelon, regeneration efficiency substantially increases when GRFs and GIFs are fused into a single chimeric construct (Debernardi *et al.*, 2020; Feng *et al.*, 2021; Bull *et al.*, 2023).

Because Arabidopsis *GRF5* has been implicated in prolongation of the cell proliferation phase and in enhancement of regeneration and transformation efficiency (Horiguchi *et al.*, 2005; Omidbakhshfard *et al.*, 2015), prioritization of the GRF5 clade was undertaken. Recent studies reported that GRF–GIF chimeras improve tomato regeneration primarily using cotyledon or hypocotyl explants (Swinnen *et al.*, 2025; Tang *et al.*, 2025). Notably, the GRF genes employed in these studies represent distinct members of the tomato GRF family, underscoring potential functional diversity among GRF paralogs. In this study, we focused on *SlGRF4* (Solyc07g041640.2), the closest tomato homolog of Arabidopsis *GRF5*, to examine whether a GRF–GIF chimera derived from this paralog could robustly enhance regeneration from true leaf explants. The tomato homologs of Arabidopsis *GRF5* and *GIF1* were identified as *SlGRF4* and *SlGIF1b*, respectively. Based on a previous report that GRF–GIF chimeras enhance regeneration in both monocots and dicots, including wheat and citrus (Debernardi *et al.*, 2020), it was hypothesized that an *SlGRF4–SlGIF1b* fusion could improve regeneration efficiency in tomato leaf explants. The establishment of an effective regeneration strategy from leaf explants was considered a means to develop a more flexible strategy for tomato transformation beyond cotyledon- or hypocotyl-based systems. In addition to the evaluation of GRF–GIF chimeras in regeneration, potential post-transcriptional and post-translational regulation mechanisms were examined. Post-transcriptional regulation of GRFs by miRNA396 has been well established (Omidbakhshfard *et al.*, 2015), but post-translational regulation of GRF–GIF chimeras (e.g. ubiquitination) has yet to be clarified. Mechanisms at the RNA level, including structural stability and decay kinetics, were also considered.

Preliminary observations indicated that GRF–GIF fusion proteins accumulated poorly in regenerated tomato tissues despite robust transcript detection, suggesting a possible post-transcriptional limitation on protein abundance. Ubiquitin-mediated proteasomal degradation is a major regulatory pathway for plant transcription factors, and multi-site ubiquitination has been shown to exert complex, non-linear effects on protein turnover (Breeze, 2020). However, the contribution of ubiquitination to the regulation of GRF–GIF chimeras has not been examined. These considerations motivated us to examine RNA abundance and protein accumulation of GRF–GIF chimeras during regeneration.

Accordingly, three objectives were pursued in this study: (i) to determine whether GRF–GIF chimeras enhance tomato leaf explant regeneration; (ii) to define the ubiquitination-linked regulation of GRF–GIF protein; and (iii) to delineate post-transcriptional contributions by assessing mRNA stability and structural features within transcriptome-wide programs.

## Materials and methods

### Plant materials and seed germination

*Solanum lycopersicum* cultivars MicroTom, Doterang, and Gwangbok were used in this study. MicroTom seeds were obtained from in-house stock; Doterang seeds (Cat. no. 2008-337) and Gwangbok seeds (Cat. no. 02-0005-2014-92) were purchased from TAKII and Aram Seed, respectively. Seeds were surface-sterilized with 70% ethanol for 1 min, then immersed in a solution containing 1% sodium hypochlorite and 0.05% Triton X-100 for 10 min. After sterilization, seeds were rinsed eight times with sterile triple-distilled water. The sterilized seeds were then incubated at 4 °C for 2 d for cold stratification prior to sowing on incubation dishes (SPL, Cat. no. 310070) that contained half-strength Murashige and Skoog (MS) medium supplemented with 20 g l^–1^ sucrose and 8 g l^–1^ phyto agar. Twelve seeds were sown on each dish, and dishes were incubated in a growth chamber at 25±0.2 °C under a 16 h light/8 h dark photoperiod.

### Molecular cloning

For DNA cloning, *AtGRF5* (At3g13960.1) and *AtGIF1* (At5g28640.1) were amplified from cDNA synthesized using total RNA extracted from germinated *Arabidopsis thaliana* (Col-0) seeds with PrimeSTAR DNA polymerase (Takara Bio Inc., Cat. no. R040A, Kyoto, Japan). Similarly, *SlGRF4* (Solyc07g041640.2.1) and *SlGIF1b* (Solyc11g006230.1.1) were amplified from MicroTom cDNA (Supplementary Table S1). After sequence verification, the stop codon was removed from *AtGRF5* and *SlGRF4*, and an alanine linker (GCGGCCGCTGCC) was introduced at the C-terminal end. Each *GRF* gene was fused with *AtGIF1* or *SlGIF1b* by overlap PCR to generate a single chimeric gene. The resulting fusion constructs were cloned into the pCAMBIA2300-1 vector. A hemagglutinin (HA) tag (TACCCATACGATGTTCCAGATTACGCT) was fused at the C-terminal end of each construct.

### Preparation of explants

Fully expanded true leaves from 20-day-old seedlings of MicroTom and Doterang were used as explants. For each leaf, the petiole and distal tip (1–2 mm) were removed, and the remaining tissue was trimmed into uniformly sized segments (∼5 mm) using a sterile scalpel. Explants collected from multiple seedlings were pooled on a sterile plate and randomly mixed prior to *Agrobacterium* inoculation to avoid positional bias. For cv. Gwangbok, cotyledons from 11-day-old seedlings were used as explants. Cotyledons were excised at the base and trimmed into uniform segments (∼5 mm) using a sterile scalpel. During pre-culture (1 d) and co-cultivation (3 d in darkness), all explants were cultured with the adaxial side facing the medium. After washing, explants were transferred to regeneration medium and cultured with the abaxial side facing the medium.

### *Agrobacterium*-mediated transformation

The GRF–GIF chimera overexpression construct was introduced into the pCAMBIA2300-1 vector and subsequently transformed into *Agrobacterium tumefaciens* strain EHA105. The *Agrobacterium* culture was grown in lysogeny broth (LB) medium (5 g l^–1^ yeast extract, 10 g l^–1^ tryptone, 10 g l^–1^ sodium chloride, pH 7.0±0.2) until it reached an optical density at 600 nm (OD_600_) of 0.6. Bacterial cells were harvested by centrifugation and resuspended in 30 ml of liquid MS medium. The prepared explants were co-cultivated with the bacterial suspension in liquid MS medium containing 100 μM acetosyringone at 120 rpm for 15 min. Excess liquid was removed by placing explants on sterile filter paper, and explants were incubated on co-culture medium [MS agar medium containing 0.2 mg l^–1^ indole-3-acetic acid (IAA), 2.0 mg l^–1^ zeatin, 50 μM acetosyringone] under dark conditions for 3 d at 25±2 °C. Following co-cultivation, explants were washed sequentially with liquid MS medium containing 750 mg l^–1^ cefotaxime for 5 min (Wash I), followed by 450 mg l^–1^ cefotaxime for 5 min (Wash II). The washed explants were transferred to shoot induction medium (SIM; MS agar medium containing 0.2 mg l^–1^ IAA, 2.0 mg l^–1^ zeatin, 100 mg l^–1^ kanamycin, and 300 mg l^–1^ carbenicillin) and cultured under long-day conditions (16 h light/8 h dark). Explants were subcultured weekly onto fresh SIM until shoot regeneration was observed.

### Shoot regeneration from tomato explants

Shoot regeneration was performed using the prepared explants cultured on SIM at 25±2 °C under a long-day photoperiod (16 h light/8 h dark). Explants were transferred to fresh SIM weekly. Shoot regeneration frequency was determined as the proportion of explants that produced visible shoots relative to the total number of explants. Root regeneration frequency was assessed by counting regenerated shoots that successfully formed roots. Shoots that reached a length of ≥1 cm were excised and transferred to rooting medium (half-strength MS agar supplemented with 20 mg l^–1^ kanamycin and 300 mg l^–1^ carbenicillin) for 2–4 weeks to promote root formation. The growth chamber was maintained at a light intensity of 60±10 µmol photons m^−2^ s^−1^. This light condition was applied consistently across all culture steps, including pre-culture, co-cultivation, and shoot and root regeneration.

### RNA isolation and quantitative reverse transcription–PCR analysis

Total RNA was extracted from MicroTom leaf explant samples collected at 0, 7, 14, 21, 28, and 35 d after treatment using the HiYield™ Total RNA Mini Kit (RBC, Cat. no. YRP100, Banqiao, Taiwan). RNA concentration and purity were determined with a NanoDrop 2000 spectrophotometer (Thermo Fisher Scientific, Waltham, MA, USA). For cDNA synthesis, 1 µg of total RNA was reverse transcribed using the EcoDry™ Premix Oligo dT Kit (Takara Bio Inc., Cat. no. 639543). A total reaction volume of 25 µl was prepared, consisting of 1 µl of cDNA template and 2× TB Green Fast qPCR Mix (Takara Bio Inc.). Reverse transcription–PCR (RT–qPCR) was performed in accordance with the manufacturer’s protocol to assess auxin-related gene expression. Amplifications were conducted on a Thermal Cycler Dice Real-Time PCR system (Takara Bio Inc.). Thermal cycling conditions included an initial denaturation step at 95 °C for 30 s, followed by 40 cycles of 95 °C for 5 s and 60 °C for 10 s. To verify product specificity, melting curve analysis was performed with dissociation steps at 95 °C for 15 s, 60 °C for 30 s, and 95 °C for 15 s. PCR efficiency was determined using the equation E=[10^(−1/slope)^−1]×100%, based on the slope of the standard curve, ensuring values within the optimal range of 90–110% (Radonić *et al.*, 2005). Relative gene expression levels were quantified using the 2^−ΔΔCt^ method and normalized to *Slactin and Sltubulin* mRNA as the internal control. Primers for GRF–GIF quantification were designed across the chimeric junction region (GRF 3′–linker–GIF 5′), which is absent from endogenous genes; thus, only the transgene was amplified. Each biological replicate consisted of a randomized pool of 4–5 full explants collected at each time point. Primers (listed in Supplementary Table S2) were designed using the GenScript Real-Time PCR (TaqMan) Primer and Probes Design Tool (https://www.genscript.com/tools/real-time-pcr-taqman-primer-design-tool), with a length of 20–26 nucleotides, target amplicon sizes between 50 bp and 500 bp, and annealing temperatures between 52 °C and 60 °C. Each RT–qPCR analysis was performed with three independent biological replicates.

### RNA sequencing

Tomato explants incubated on SIM for 0, 14, and 28 d were used for RNA sequencing analysis. Library construction was performed using 1 μg of total RNA with the TruSeq Stranded mRNA Prep Kit (Illumina, San Diego, CA, USA; Cat. no. 20020595). Raw sequencing data were generated on the Illumina NovaSeq X Plus platform. Adapter trimming, low-quality base filtering, and read alignment were performed in CLC Genomics Workbench v23.2.0 (QIAGEN, Aarhus, Denmark); the resulting clean reads were mapped to the *S. lycopersicum* cv. MicroTom reference genome sequence from the MicroTom database (https://doi.org/10.6084/m9.figshare.21509817). To identify genes with differential expression between the control and experimental groups, analysis was conducted using DESeq2 software (v.1.30.1) (Love *et al.*, 2014). Genes with low expression (median <5 counts in both groups) were excluded. Pairwise comparisons were then performed between each experimental group and the control (empty vector [EV]) at each time point (days 0 [D0], 14 [D14], and 28 [D28]). Differentially expressed genes (DEGs) were defined as those with an adjusted *P*-value (*P*_adj_)<0.01 and a |log_2_ fold change (FC)| ≥1.

To characterize temporal expression patterns, DEGs were classified into eight categories based on the direction of log_2_FC values across the three time points. Genes within each pattern group were aggregated, and heatmaps and cluster plots were generated to visualize expression profiles. To investigate the functions of pattern-matched genes, DEGs were compared between *A. thaliana* and *S. lycopersicum* constructs. Genes exhibiting identical temporal patterns in both species were identified; subsets corresponding to up-regulated patterns (patterns: + + +, + + −, − + −, − + +) and down-regulated patterns at the D14 time point were analyzed separately. Functional enrichment analysis was performed on these gene sets using the clusterProfiler (v.4.16.0) (Wu *et al.*, 2021) package, and Gene Ontology (GO) terms derived from the MicroTom database served as the annotation reference.

### *In vivo* ubiquitination assay of GRF–GIF chimera proteins

To examine the *in vivo* ubiquitination status of GRF–GIF chimera proteins, *A. tumefaciens* strain EHA105 harboring 35S:*AtGRF5–GIF1*-HA or 35S:*SlGRF4–GIF1b*-HA was cultured in LB medium supplemented with the appropriate antibiotics at 28 °C for 2 d. After the initial incubation, the bacterial culture was transferred to fresh LB medium containing four times the original antibiotic concentration and grown until it reached an OD_600_ of 0.4. Cells were collected by centrifugation and resuspended in infiltration buffer, comprising 10 mM MES (pH 5.6), 10 mM MgCl_2_, and 100 µM acetosyringone. Transient expression was carried out in *Nicotiana benthamiana* leaves, and plants were maintained under standard light conditions. To inhibit protein degradation, 100 µM MG132 (Enzo Life Sciences, Farmingdale, NY, USA) was infiltrated 16 h before sampling. Three days post-infiltration, total proteins were extracted using a lysis buffer containing 50 mM Tris-MES (pH 8.0), 0.5 M sucrose, 1 mM MgCl_2_, 10 mM EDTA, 5 mM DTT, and a complete protease inhibitor cocktail (Roche, Indianapolis, IN, USA). For immunoprecipitation, crude extracts were incubated with Pierce™ Anti-HA magnetic beads (Thermo Scientific) at 4 °C for 16 h. Samples were separated via 10% SDS–PAGE and subjected to immunoblotting using an anti-HA tag monoclonal antibody (Invitrogen, Carlsbad, CA, USA) and an anti-UBQ11 antibody (Agrisera, Vännäs, Sweden). The same experimental procedures were utilized to assess protein stability in the following *AtGRF5–GIF1* mutants: AtK363R, AtK605R, and AtK363 605R, as well as *SlGRF4–GIF1b* mutants: SlK150R, SlK205R, SlK564R, and SlK150 205 564R.

### mRNA stability analysis

#### RNA secondary structure prediction

Coding sequences of AtGRF5–GIF1 and SlGRF4–GIF1b fusion constructs were subjected to mRNA secondary structure production using RNAfold (http://rna.tbi.univie.ac.at). Minimum free energy (MFE) structures were computed at 37 °C with default parameters.

#### mRNA stability assay

For *in vivo* mRNA decay analysis, GRF–GIF constructs were transiently expressed in *N. benthamiana* leaves. Three days post-infiltration, the leaves were treated with 80 µM actinomycin D (Sigma-Aldrich, St. Louis, MO, USA) to inhibit *de novo* transcription. Leaf samples were collected at 0, 2, 4, 6, and 8 h after treatment. Total RNA was extracted and analyzed by RT–qPCR, as previously described. Relative mRNA levels at each time point were normalized to the 0 h value and fitted to a one-phase exponential decay model to estimate mRNA half-life (*t*_1/2_). The decay constant (*k*) was calculated using the following equation: *k*=ln(2)/*t*_1/2_. Decay curves were generated to compare transcript stability among constructs.

### Phylogenetic analysis

GRF and GIF protein sequences from tomato (*S. lycopersicum*) were retrieved from the National Center for Biotechnology Information (https://www.ncbi.nlm.nih.gov/) and Sol Genomics Network (https://solgenomics.net/) databases. Multiple sequence alignment of GIF proteins was performed using ClustalX 2.1 (Larkin *et al.*, 2007), and a phylogenetic tree was constructed in MEGA (v.11) (Tamura *et al.*, 2021) using the Neighbor–Joining method with 1000 bootstrap replicates.

### Multiple sequence alignment and conserved domain analysis

GRF and GIF protein sequences from tomato (*S. lycopersicum*), Arabidopsis (*A. thaliana*), rice (*Oryza sativa*), and maize (*Zea mays*) were aligned using ClustalX 2.1 and visualized with GeneDoc software. Conserved regions were identified, and three key domains—QLQ, WRC, and SSXT—were manually annotated based on sequence conservation and previous studies. These domains were marked with red boxes to highlight conserved residues across species. Alignment results were analyzed to determine sequence similarity and conservation, providing insights into the structural and functional roles of GRF and GIF proteins.

### Statistical analysis

For all experiments, statistical analyses were performed using either Student’s *t*-test or one-way ANOVA followed by Sidak’s multiple comparisons test, depending on the number of groups compared. Differences were considered statistically significant for *P*-values <0.05. All experiments were conducted with at least three biological replicates.

## Results

### Identification of *AtGRF5* and *AtGIF1* homologs in tomato

To investigate whether the GRF–GIF complex enhances shoot regeneration in tomato, we identified homologs of *AtGRF5* and *AtGIF1* in *S. lycopersicum*. A phylogenetic analysis was conducted using GRF and GIF protein sequences from *A. thaliana*, *O. sativa*, *Z. mays*, and *S. lycopersicum* (Supplementary Fig. S1). The results indicated that *SlGRF4* is the closest homolog of *AtGRF5*, exhibiting high sequence similarity, particularly in the QLQ (glutamine–leucine–glutamine) and WRC (tryptophan–arginine–cysteine) domains, which are essential for transcriptional regulation and protein–protein interactions. Similarly, *SlGIF1b* was identified as the tomato homolog of *AtGIF1*, sharing a conserved SSXT domain that mediates interaction with GRF proteins.

To confirm functional conservation, multiple sequence alignment analysis was performed (Supplementary Fig. S2). The results revealed a high degree of conservation between *AtGRF5* and *SlGRF4*, particularly within the QLQ domain, which facilitates GRF–GIF interaction, and the WRC domain, which mediates DNA binding. Such conservation suggests that *SlGRF4* retains the transcriptional regulatory functions of *AtGRF5*. Similarly, *SlGIF1b* exhibited a conserved SSXT domain, supporting its functional equivalence to *AtGIF1* in mediating GRF–GIF interactions. These results are consistent with previous findings (Khatun *et al.*, 2017) and were validated through independent amino acid sequence analysis in the present study.

Given this conservation, we hypothesized that *SlGRF4* and *SlGIF1b* could functionally replace *AtGRF5* and *AtGIF1* in tomato regeneration studies. To test this hypothesis, we generated an *SlGRF4–GIF1b* fusion construct with an alanine (A) linker, in accordance with the strategy described by Debernardi *et al.* (2020); we also established a homologous *AtGRF5–AtGIF1* fusion construct. These constructs were used in subsequent experiments to assess their effects on shoot regeneration efficiency in tomato leaf explants.

### GRF–GIF enhances regeneration efficiency in tomato

**Fig. 1.**
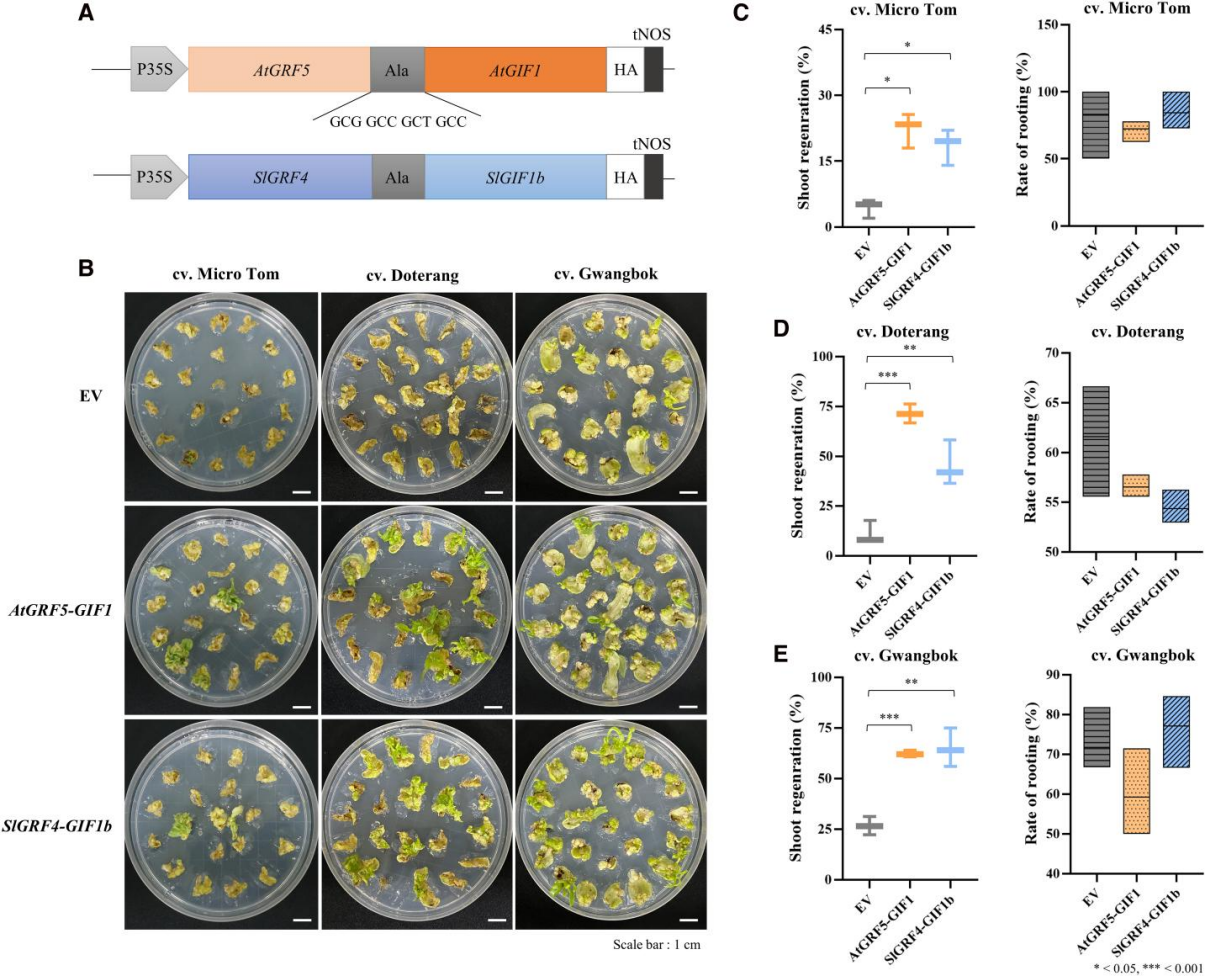
The GRF–GIF chimera enhances shoot regeneration efficiency in tomato explants. (A) Schematic representation of the *AtGRF5* (pale orange)–*GIF1* (orange) and *SlGRF4* (sky)–*GIF1b* (blue) constructs used in this study. The constructs consist of *Arabidopsis thaliana GRF5* (*AtGRF5*) or *Solanum lycopersicum GRF4* (*SlGRF4*) fused to *A. thaliana GIF1* (*AtGIF1*) or *S. lycopersicum GIF1b* (*SlGIF1b*), respectively, with a C-terminal HA tag under the control of the CaMV 2×35S promoter. (B) Representative images of tomato leaf explants at 28 d post-transformation, showing shoot regeneration in plants transformed with the empty vector (EV), *AtGRF5–GIF1*, and *SlGRF4–GIF1b*. Scale bar, 1 cm. Each experiment included 50–70 explants. (C) Shoot regeneration efficiency in three tomato cultivars: MicroTom, Doterang, and Gwangbok. Regeneration efficiency was calculated as the percentage of explants producing shoots relative to the total number of explants (mean ±SD; *n*=3 biological replicates). (D) Rooting efficiency of regenerated shoots in the three tomato cultivars. Box plots represent variation in rooting rates among independent transformation events; the median is indicated by the center line. Statistical significance was determined using Student’s *t*-test (*P<0.05; **P<0.01; ***P<0.001).

To evaluate the effect of GRF–GIF chimeric proteins on tomato regeneration, *AtGRF5–GIF1* and *SlGRF4–GIF1b* constructs under the cauliflower mosaic virus (CaMV) 2×35S promoter were introduced into three cultivars—MicroTom, Doterang, and Gwangbok (Fig. 1A). For MicroTom and Doterang, leaf explants were deliberately selected, rather than the more commonly used cotyledon or hypocotyl explants (Sandhya *et al.*, 2022), to increase the number of explants obtainable per donor plant and to test whether GRF–GIF could enhance regeneration from a tissue with relatively lower baseline competence. This design allowed us to test GRF–GIF in a more regeneration-limited yet practically high-explant-yield explant system. In contrast, cotyledon explants were used for Gwangbok, given that leaf explants exhibited insufficient responsiveness under our conditions. To confirm that all three cultivars were susceptible to *Agrobacterium*-mediated gene delivery, we performed transient cotyledon infiltration using a 35S:eGFP construct and detected enhanced green fluorescent protein (eGFP) in MicroTom, Doterang, and Gwangbok by immunoblotting (Supplementary Fig. S3). After *Agrobacterium*-mediated transformation, shoot regeneration rates were monitored in plants cultured on regeneration medium. Both GRF–GIF constructs significantly enhanced shoot regeneration efficiency compared with the EV control (Fig. 1B). In MicroTom, the regeneration rate increased from 4.39% in the EV to 22.34% in *AtGRF5–GIF1* and to 18.51% in *SlGRF4–GIF1b* lines (Fig. 1C; Supplementary Table S3). The effect was even more pronounced in Doterang and Gwangbok. In Doterang, regeneration efficiency increased 6.4-fold in *AtGRF5–GIF1* and 4.11-fold in *SlGRF4–GIF1b* compared with the EV, reaching 76.3% and 58.3%, respectively (Fig. 1D; Supplementary Table S3). In Gwangbok, regeneration rates were 26.7% in the EV, whereas *AtGRF5–GIF1* and *SlGRF4–GIF1b* lines exhibited ∼2.3-fold increases, reaching 62.2% and 65%, respectively (Fig. 1E; Supplementary Table S3). Statistical analysis confirmed that both GRF–GIF constructs significantly improved regeneration efficiency (*P*<0.01).

Given that successful rooting is critical for plant establishment, we analyzed rooting efficiency in regenerated shoots across all three cultivars to determine developmental stability. Root formation was consistently observed in MicroTom, Doterang, and Gwangbok, indicating that regenerated shoots retained viability and the capacity to progress to the next developmental stage. Root formation was consistently achieved in all cultivars examined, suggesting that the regenerated shoots possessed intact developmental competence throughout the regeneration and rooting phases.

Our findings suggest that the GRF–GIF chimeric protein plays a crucial role in enhancing shoot regeneration by promoting cell proliferation and meristematic tissue reprogramming in explants, regardless of the tomato cultivar. Additionally, the high rooting efficiency indicates that regenerated shoots successfully progressed to the next stage of plant development, reinforcing the potential of GRF–GIF-mediated strategies to improve transformation efficiency in tomato and other recalcitrant crops. In addition to the enhanced regeneration phenotype, GRF–GIF transgenic plants exhibited normal vegetative growth and fertility at both the T_0_ and T_1_ generations (Supplementary Fig. S4).

### Quantitative measurement of gene expression

**Fig. 2.**
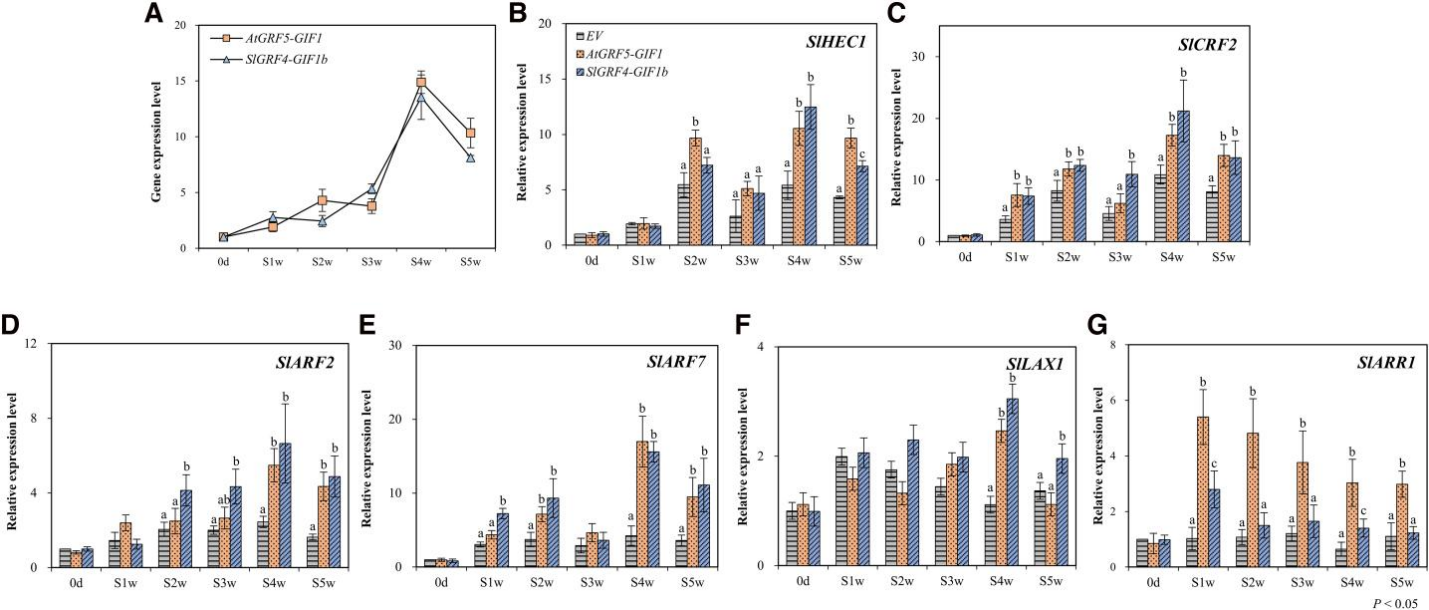
Temporal expression analysis of GRF–GIF fusion constructs and their downstream targets during shoot regeneration. (A) Gene expression of *AtGRF5–GIF1* and *SlGRF4–GIF1b* was analyzed in T_0_ transgenic MicroTom leaf explants at 0, 1, 2, 3, 4, and 5 weeks post-transformation. (B–G) Transcript levels of *HEC1*, *CRF2*, *ARF2*, *ARF7*, *LAX1*, and *ARR1* were analyzed in explants collected at 0, 1, 2, 3, 4, and 5 weeks post-transformation to examine the downstream effects of GRF–GIF expression. Primers used to measure GRF–GIF transcripts span the GRF–linker–GIF junction, ensuring transgene-specific amplification. Each RT–qPCR sample consisted of a randomized pool of 4–5 explants per biological replicate. Data are presented as the mean ±SD (*n*=3). Statistical significance was determined using one-way ANOVA followed by Sidak’s multiple-comparison test; groups that do not share a letter differ significantly at *P*<0.05. Comparisons without letters were not statistically significant.

To investigate the transcriptional dynamics of GRF–GIF constructs, RT–qPCR was performed at 0, 1, 2, 3, 4, and 5 weeks post-transformation (Supplementary Fig. S5; Supplementary Tables S4, S5). Both *AtGRF5–GIF1* and *SlGRF4–GIF1b* transgenic lines exhibited gradual increases in transcript accumulation, peaking at week 4 before declining at week 5 (Fig. 2A). This temporal expression pattern closely corresponded with the observed regeneration phenotypes: callus formation began around week 2, and maximal shoot emergence occurred between weeks 3 and 4. These findings indicate that GRF–GIF expression is tightly correlated with the developmental timing of regeneration. To determine how GRF–GIF expression influences early regeneration and meristem initiation, we quantified the expression levels of six genes involved in hormonal signaling and meristem function: *HECATE1* (*HEC1*), *CYTOKININ RESPONSE FACTOR 2* (*CRF2*), *AUXIN RESPONSE FACTOR 2* (*ARF2*), *AUXIN RESPONSE FACTOR 7* (*ARF7*), *Like-AUX1 1* (*LAX1*), and *ARABIDOPSIS RESPONSE REGULATOR 1* (*ARR1*). Among these, *SlHEC1* and *SlCRF2*—genes associated with meristem maintenance and cytokinin signaling—were strongly up-regulated in both GRF–GIF lines; peak expression occurred at week 4, coinciding with transgene expression (Fig. 2B, C). Similarly, *SlARF2* and *SlARF7* expression gradually increased and peaked at week 4 before declining (Fig. 2D, E). *SlLAX1*, encoding an auxin influx carrier, also displayed its highest expression at week 4 (Fig. 2F), consistent with active shoot formation. Intriguingly, *SlARR1*, encoding a cytokinin response regulator involved in callus initiation, showed distinct temporal dynamics: *AtGRF5–GIF1* induced an early peak at week 1, whereas *SlGRF4–GIF1b* exhibited a delayed peak at week 3 (Fig. 2G), followed by a gradual decline.

### RNA sequencing

**Fig. 3.**
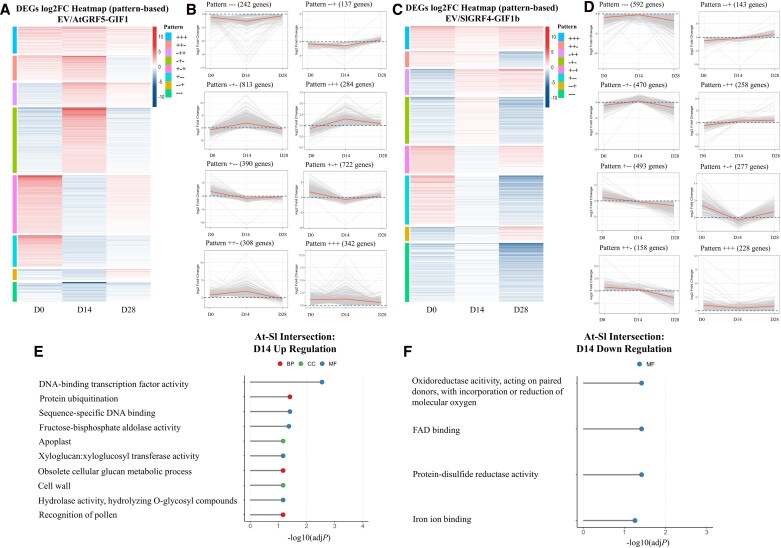
RNA sequencing of GRF–GIF-expressing tomato leaf explants. (A, C) Heatmaps of log_2_ fold change (log_2_FC) for differentially expressed genes (DEGs) in explants overexpressing *AtGRF5–GIF1* (A) or *SlGRF4–GIF1b* (C) versus empty vector (EV) at day 0 (D0), day 14 (D14), and day 28 (D28). Genes were grouped into eight temporal expression patterns. (B, D) Line plots showing log_2_FC trajectories for each pattern (gray=individual genes, red=mean, dashed blue=log_2_FC of 0). Numbers indicate gene counts per pattern. (E, F) Gene Ontology (GO) terms enriched in DEGs commonly up-regulated (E) or down-regulated (F) at D14 in both constructs.

Because the quantitative expression analysis (Fig. 2) demonstrated that regeneration-related regulators reached maximal induction at week 4 (D28), transcriptome profiling focused on the preceding D14 stage to capture early reprogramming events driven by GRF–GIF overexpression. RNA sequencing was performed for EV, *AtGRF5–GIF1*, and *SlGRF4–GIF1b* explants collected at D0, D14, and D28 (three biological replicates per condition). After quality control and normalization, shrunken log_2_FC values (EV versus each construct at each time point) were calculated; DEGs (|log_2_FC|≥1, *P*_adj_<0.01) were clustered according to their temporal log_2_FC trajectories. Eight distinct expression patterns (+ + +, + + –, – + +, – + –, + – +, + – –, – –+, and – – –) were identified in both comparisons (Fig. 3A, C). A pronounced transcriptional shift at D14 was observed in both GRF–GIF lines, and *AtGRF5–GIF1* generally exhibited greater FC amplitudes (Fig. 3B, D; Supplementary Table S6).

To define a core program shared by the two constructs, genes assigned to equivalent temporal patterns in both comparisons were selected. Genes exhibiting D14-specific up-regulation (+ + +, + + –, – + +, – + –; *n*=240 total) were merged into a single D14-up union, and those showing D14-specific down-regulation (+ – +, + – –, – – +, – – –; *n*=221 total) were merged into a D14-down union (detailed gene counts are provided in Supplementary Table S7). These union sets were subjected to GO term over-representation analysis. Enrichment of the D14-up union revealed terms associated with DNA-binding transcription factor activity, protein ubiquitination and proteostasis, and cell wall and extracellular remodeling, indicating a strong early transcriptional and structural reprogramming response (Fig. 3E; Supplementary Table S8). In contrast, the D14-down union was enriched for oxidoreductase activity and cofactor binding functions, suggesting coordinated reduction of redox-related metabolism during this transition (Fig. 3F; Supplementary Table S8).

Collectively, these results indicate that both GRF–GIF constructs initiate a shared early (D14) transcriptional reprogramming program characterized by activation of transcriptional control and proteostasis pathways and concurrent repression of redox-metabolic functions. The enrichment of protein ubiquitination-related terms in the D14-up-regulated set aligns with the ubiquitin-dependent regulation of GRF–GIF protein stability demonstrated in later biochemical assays, linking the early transcriptomic response to the post-translational control of GRF–GIF activity.

### Ubiquitination of GRF–GIF chimeric proteins in transient expression assays

**Fig. 4.**
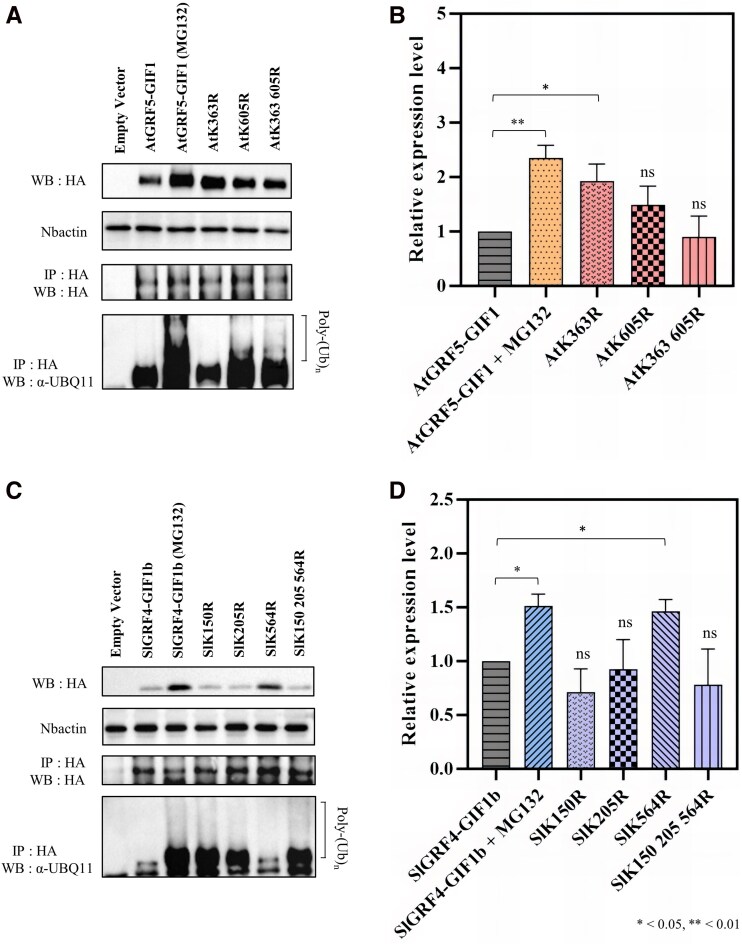
Identification of ubiquitination sites regulating GRF–GIF protein stability in transient expression assays. (A) Western blot analysis of AtGRF5–GIF1 protein accumulation. HA-tagged AtGRF5–GIF1 was transiently expressed in *N. benthamiana* leaves. MG132 (100 µM, 24 h) treatment was applied to assess proteasome involvement in degradation. Co-immunoprecipitation (Co-IP) assays with anti-HA and anti-ubiquitin antibodies confirmed ubiquitination. (B) Quantification of AtGRF5–GIF1 protein levels. Protein levels were normalized to actin and are shown as the mean ±SD (*n*=3). The K363R mutation significantly increased protein stability, whereas the K363 605R double mutation did not further enhance accumulation. (C) Western blot analysis of SlGRF4–GIF1b protein accumulation. HA-tagged SlGRF4–GIF1b was transiently expressed in *N. benthamiana*. MG132 treatment and Co-IP assays were performed as described in (A). (D) Quantification of SlGRF4–GIF1b protein levels. The K564R mutation stabilized SlGRF4–GIF1b, whereas the K150 205 564R triple mutation showed no additional increase. Data are presented as the mean ±SD (*n*=3). Statistical significance was determined using Student’s *t*-test (*P<0.05; **P<0.01).

Post-translational modifications, particularly ubiquitination, serve as key regulatory mechanisms that control protein stability in plants. Although ubiquitination has been identified as a major process regulating the stability of multiple transcription factors, its role in the regulation of GRF and GIF proteins has remained unclear. Consistent with this possibility, our RNA-seq analysis revealed that several genes associated with protein ubiquitination were up-regulated during GRF–GIF-induced regeneration, suggesting that post-translational regulation may contribute to the activity of GRF–GIF chimeras. In this study, we examined whether the GRF–GIF chimeric protein undergoes ubiquitination and proteasomal degradation in tomato. During initial experiments, transgenic tomato plants expressing GRF–GIF fusion constructs exhibited enhanced shoot regeneration efficiency. However, western blot analysis failed to detect GRF–GIF proteins in regenerated transgenic plants, despite clear phenotypic effects and RT–PCR-confirmed transgene integration (Supplementary Fig. S6). This discrepancy suggested that GRF–GIF proteins undergo rapid degradation. To test this hypothesis, we performed transient expression assays in *N. benthamiana* leaves. GRF–GIF protein levels substantially increased after treatment with MG132, a proteasome inhibitor, indicating degradation via the ubiquitin–proteasome system (Fig. 4). To confirm the presence of ubiquitination-mediated degradation, we conducted co-immunoprecipitation (Co-IP) assays using anti-HA antibodies in *N. benthamiana*. Immunoblots probed with anti-ubiquitin antibodies revealed higher molecular weight ubiquitinated forms of AtGRF5–GIF1 and SlGRF4–GIF1b, confirming modification by the ubiquitin pathway (Fig. 4A, C; Supplementary Fig. S7).

To identify potential ubiquitination sites within GRF–GIF fusion proteins, we performed UbPred analysis and selected residues with prediction scores >0.84 (Supplementary Table S9). For AtGRF5–GIF1, lysine residues at positions 363 and 605 were substituted with arginine (K363R, K605R), whereas SlGRF4–GIF1b carried substitutions at positions 150, 205, and 564 (K150R, K205R, K564R) (Table 1). Additionally, multi-site mutation constructs incorporating all predicted ubiquitination sites were generated for both fusions. Western blot analysis revealed that the AtK363R construct exhibited no detectable ubiquitination, and its protein accumulation was comparable with that observed in MG132-treated *AtGRF5–GIF1* plants (Fig. 4B; Supplementary Table S10; Supplementary Fig. S8). Furthermore, SlGRF4–GIF1b, which generally exhibited lower protein expression than AtGRF5–GIF1, also showed increased protein stability after site-directed mutagenesis. The SlK564R construct displayed protein accumulation levels comparable with those of MG132-treated samples, indicating that K564 serves as a key ubiquitination site influencing SlGRF4–GIF1b accumulation (Fig. 4D; Supplementary Table S10; Supplementary Fig. S8). Intriguingly, plants expressing multi-site mutation constructs (AtK363 605R, SlK150 205 564R) did not exhibit further increases in protein stability, suggesting the existence of compensatory mechanisms regulating GRF–GIF protein levels. Similar results have been reported for other plant proteins, where multi-site ubiquitination mutations do not always lead to proportional reductions in degradation rates (Romero-Barrios and Vert, 2018). The identification of key ubiquitination sites provides new insights into post-translational mechanisms controlling GRF–GIF stability.

**Table 1.** The ubiquitination prediction tool UbPred was used to identify potential lysine (K) residues targeted for ubiquitination in GRF–GIF fusion proteins

Gene names	Ub sites	Score	Forecast tool	Nucleotide sequence	Modified sequence
AtGRF5 (NM_112250.3)	K363	0.84	UbPred	TCC**AAG**AAG	TCC**AGG**AAG
AtGIF1 (NM_122747.4)	K204	0.88	UbPred	TTG**AAA**TCA	TTG**AGA**TCA
SlGRF4 (XM_004243669.4)	K150	0.91	UbPred	AAC**AAA**AAC	AAC**AGA**AAC
SlGRF4 (XM_004243669.4)	K205	0.98	UbPred	ATG**AAA**GAG	ATG**AGA**GAG
SlGIF1b (XM_004249872.4)	K216	0.85	UbPred	TTG**AAA**TCT	TTG**AGA**TCT

Lysine residues with a score ≥0.62 were considered significant ubiquitination sites. Based on this analysis, AtGRF5 K363, AtGIF1 K204, SlGRF4 K150, SlGRF4 K205, and SlGIF1b K216 were identified as candidate ubiquitination sites. These residues were selected for mutational analysis due to their high prediction scores, indicating a strong likelihood of ubiquitin conjugation. The original data, including complete lists of predicted ubiquitination sites, are provided in Supplementary Table S9.

**Fig. 5.**
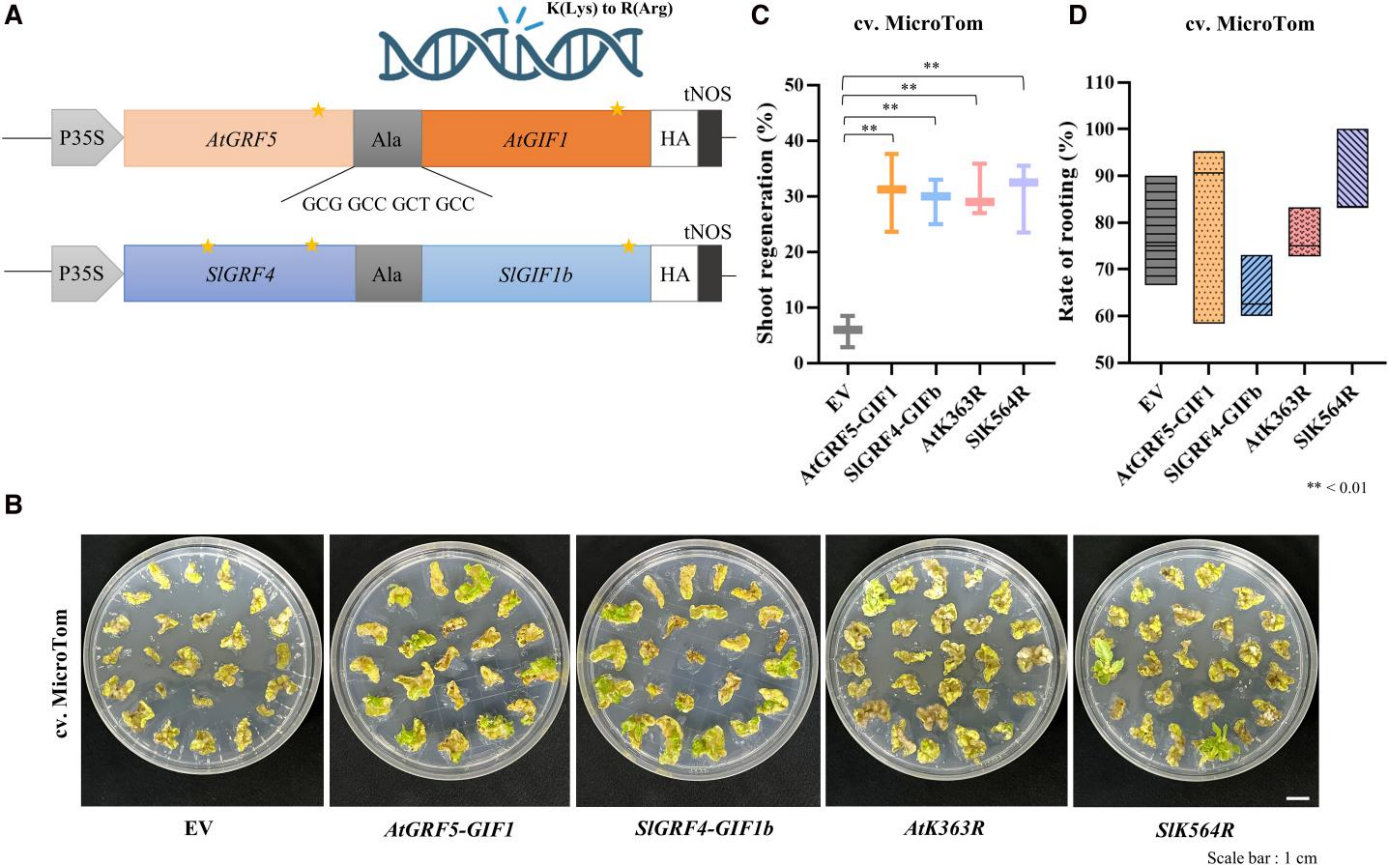
GRF–GIF point mutation chimeras improve shoot regeneration efficiency in tomato explants. (A) Schematic representation of HA-tagged chimeras driven by the 35S promoter: *AtGRF5–AtGIF1* and *SlGRF4–SlGIF1b*. Yellow stars indicate Lys→Arg substitutions at UbPred-predicted ubiquitination sites (AtK363R and SlK564R). (B) Representative plates of leaf explants cultured on SIM (left to right: EV, wild-type chimeras, AtK363R, and SlK564R). (C) Shoot regeneration frequency per explant (%; box-and-whisker plot). (D) Rooting frequency (%) of regenerated shoots, summarized as in (C). Statistical significance was determined using Student’s *t*-test (**P<0.01). Both wild-type and mutant chimeras enhanced regeneration relative to EV; point-mutated forms (AtK363R and SlK564R) showed efficiency comparable with or higher than that of unmodified constructs.

To determine whether stabilization observed in transient assays could further improve regeneration, ubiquitination site mutant constructs (AtK363R and SlK564R) were stably introduced into tomato. Regeneration efficiencies were consistently higher in these lines than in the EV; however, no statistically significant differences were observed relative to plants transformed with the wild-type GRF–GIF constructs (Fig. 5; Supplementary Table S11). These findings suggest that although protein stability can be increased by disruption of individual ubiquitination sites, additional improvements in regeneration efficiency beyond that conferred by GRF–GIF expression were not achieved.

### mRNA secondary structure and decay kinetics differ between GRF–GIF constructs

**Fig. 6.**
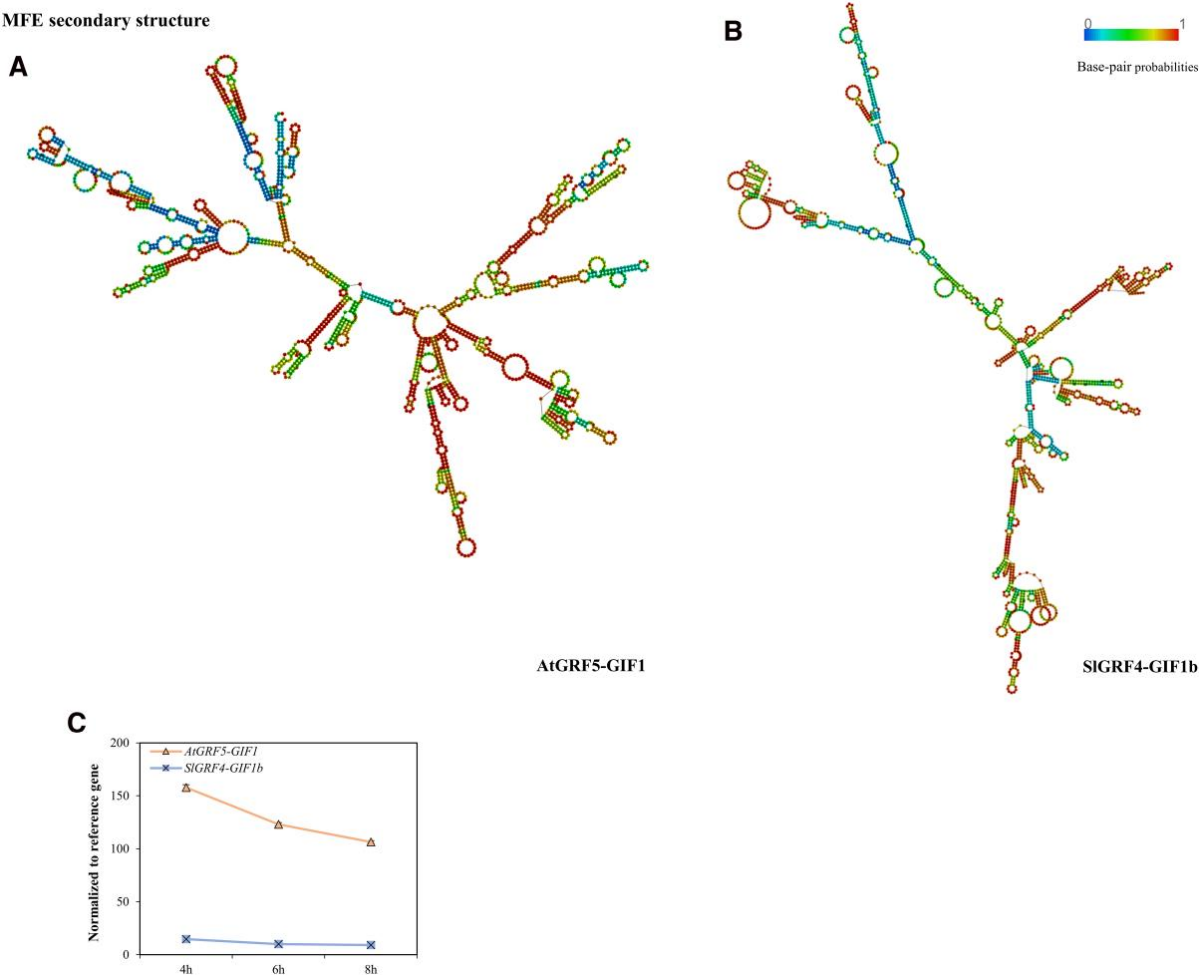
Analysis of mRNA stability of GRF–GIF constructs. (A) Summary of *in silico*-predicted structural and thermodynamic properties of *AtGRF5–GIF1* and *SlGRF4–GIF1b* mRNAs, including minimum free energy (MFE, Δ*G*), average Δ*G*, ensemble diversity, GC content, decay rate, and half-life (*t*_1/2_). (B) Predicted RNA secondary structures visualized using ViennaRNA Web Services, with base-pairing probabilities color-coded from 0 (blue) to 1 (red). (C) Experimental decay curves of GRF–GIF mRNAs in *N. benthamiana* leaves after treatment with 80 μM actinomycin D at 3 d post-infiltration. Relative mRNA levels were measured at 0, 2, 4, 6, and 8 h by RT–qPCR and fitted to a one-phase decay model. Values represent the mean ±SD (*n*=3).

Predicted mRNA secondary structures were obtained using RNAfold. *AtGRF5–GIF1* exhibited a lower MFE and reduced ensemble diversity compared with *SlGRF4–GIF1b*, indicating a thermodynamically more stable structural ensemble (Table 2; Fig. 6A, B). The predicted structure of *AtGRF5–GIF1* contained longer continuous stem–loop regions with higher GC content, whereas *SlGRF4–GIF1b* included larger internal loops and unpaired regions, features generally associated with faster decay and lower stability.

**Table 2.** Comparison of mRNA structural stability parameters for *AtGRF5–GIF1* and *SlGRF4–GIF1b*

	Construct names	
	AtGRF5–GIF1	SlGRF4–GIF1b
MFE Δ*G* (kcal mol^–1^)	−564.30	−441.10
Average Δ*G* (top 5 structures)	−561.58	−440.22
Ensemble diversity	389.58	423.86
GC contents (%)	45.68	42.41
Decay rate (h^−1^)	0.0961	0.1655
mRNA half-life (*t*_1/2_) calculation (h)	7.21	4.19

Minimum free energy (MFE), average Δ*G*, ensemble diversity, decay rate, and mRNA half-life values were calculated using RNAfold. Lower MFE and average Δ*G* values indicate higher mRNA stability, whereas greater ensemble diversity reflects increased structural variability.

To experimentally validate these predictions, transcriptional shutoff assays were performed in *N. benthamiana* leaves transiently expressing each construct (80 µM actinomycin D, 3 d post-infiltration). Normalized transcript abundance over time was fitted to a one-phase exponential model; decay constants and half-life were determined (Fig. 6C; Supplementary Table S12). The data revealed significantly slower mRNA decay for *AtGRF5–GIF1* (*k*=0.0961 h^−1^; *t*_1/2_=7.21 h) than for *SlGRF4–GIF1b* (*k*=0.1655 h^−1^; *t*_1/2_=4.19 h). These half-life values are summarized in Table 2. Taken together, the structural features and experimental decay measurements consistently support greater mRNA stability for *AtGRF5–GIF1*.

## Discussion

Efficient plant regeneration is an essential requirement for crop transformation and improvement, but regeneration efficiency remains a major limitation in tomato. In this study, GRF–GIF chimeric proteins significantly enhanced regeneration from leaf explants (Fig. 1), providing new insight into the molecular basis of cellular reprogramming and revealing a previously unrecognized regulatory layer through ubiquitination. Previous research has demonstrated that GRF–GIF modules enhance regeneration in several plant species, including Arabidopsis, lettuce, and watermelon (Debernardi *et al.*, 2020; Feng *et al.*, 2021; Bull *et al.*, 2023). Consistent with these reports, regeneration efficiency was improved in tomato by both *AtGRF5–GIF1* and *SlGRF4–GIF1b* (Fig. 1), although the magnitude of improvement varied among cultivars; the strongest effect was observed in Doterang (Fig. 1D). These genotype-specific responses probably reflect intrinsic differences in endogenous regeneration capacity (Ikeuchi *et al.*, 2016; Prihatna *et al.*, 2019), underscoring the importance of host genetic background when utilizing developmental regulators for transformation.

While recent studies have shown that GRF–GIF chimeras improve tomato regeneration primarily using cotyledon or hypocotyl explants, the present study extends these findings to true leaf explants, which are generally regarded as more abundant yet regeneration-recalcitrant tissues. Leaf explants are advantageous for transformation workflows because a larger number of explants can be obtained per donor plant and because their use is less strictly constrained by developmental stage. However, their application has been limited by low regenerative competence and unstable shoot conversion. Our results show that both *AtGRF5–GIF1* and *SlGRF4–GIF1b* substantially enhance shoot regeneration from tomato leaf explants and that this improvement is reproducible across cultivars, including MicroTom and Doterang (Fig. 1B–E). These findings demonstrate that GRF–GIF chimeras can overcome intrinsic limitations of leaf-based regeneration systems and thereby broaden the practical applicability of GRF–GIF-assisted transformation beyond previously reported explant types.

In this study, we quantified the temporal expression patterns of several regeneration-related genes to understand how GRF–GIF activity coordinates cellular reprogramming (Fig. 2). The selection of *HEC1*, *CRF2*, *ARF2*, *ARF7*, and *LAX1* was based on prior evidence that GRF/GIF complexes directly or indirectly regulate these pathways in other plant species. For example, AtGIF1/AN3 directly activates *HEC1* and *CRF2*, two key regulators of meristem maintenance and cytokinin signaling (Vercruyssen *et al.*, 2014), while *OsGRF6* binds to CGSMR motifs in the promoters of *ARF2* and *ARF7*, activating auxin-response pathways essential for organ initiation (Gao *et al.*, 2015). Although LAX1 has not been reported as a direct GRF/GIF target, it plays a central role in auxin influx and primordium initiation. LAX1 contributes to auxin accumulation at incipient meristematic sites and is essential for lateral organ formation and regeneration competence (Swarup and Péret, 2012). This role fits within the broader hormonal framework of *de novo* shoot organogenesis described in recent regeneration studies (Shin *et al.*, 2020). Its temporal induction at week 4 in our GRF–GIF lines therefore aligns with the timing of shoot initiation and is consistent with increased meristematic activity (Fig. 2F). Earlier induction of *ARR1* was observed in *AtGRF5–GIF1* lines compared with *SlGRF4–GIF1b* (Fig. 2G), but comparable regeneration efficiencies were ultimately achieved, indicating that timing differences fine-tune the regenerative response without altering final outcomes. Importantly, explants were sampled as randomized pools rather than callus-enriched tissues, and primers were designed to specifically amplify the GRF–GIF junction sequence, avoiding endogenous GRF or GIF loci. Overall, these considerations support the interpretation that the observed transcriptional dynamics reflect underlying biological activation rather than non-specific 35S promoter artifacts. Together, these findings support a model in which GRF–GIF complexes promote regeneration partly by reinforcing conserved cytokinin–auxin regulatory modules that orchestrate meristem initiation.

A shared early transcriptional response at D14 was detected in both GRF–GIF constructs (Fig. 3A–D), characterized by significant enrichment of DNA-binding transcription factor activity and sequence-specific DNA binding, consistent with transcription factor-driven acquisition of regenerative competence and *de novo* organ formation (Ikeuchi *et al.*, 2016). Protein ubiquitination was also enriched in the D14-up-regulated set (Fig. 3E), indicating activation of proteostatic pathways at reprogramming onset (Callis, 2014). In contrast, the D14-down-regulated set featured oxidoreductase-linked and cofactor binding activities—including FAD binding, protein disulfide reductase activity, and iron ion binding—that were reduced (Fig. 3F), suggesting transient attenuation of cofactor-dependent metabolism during early identity resetting before outgrowth (Kobayashi and Nishizawa, 2012; Strasser, 2020). Collectively, these findings support a framework in which early transcription factor-driven reprogramming at D14 precedes subsequent activation of downstream hormonal and developmental regulators.

GRF–GIF chimeras differed not only in transcriptional activity but also in RNA and protein stability, highlighting a multilayered regulatory framework that appears to influence regeneration efficiency. Although GRF–GIF transcripts were readily detected during regeneration, the corresponding fusion proteins were not detectable in regenerated tomato tissues, and protein ubiquitination-related terms were enriched in the RNA-seq datasets from both constructs, providing the rationale for focusing on ubiquitin-mediated regulation of GRF–GIF stability (Figs 4, 6).

Ubiquitin-mediated proteasomal degradation is a well-established process for modulating transcription factor abundance and activity in plants (Moon *et al.*, 2004; Vierstra, 2009; Nelson and Millar, 2015). Although direct ubiquitination of GRFs had not previously been reported, several lines of evidence in this study support the conclusion that GRF–GIF chimeras are degraded through the ubiquitin–proteasome system (Fig. 4). Protein accumulation increased upon proteasome inhibition (Fig. 4A, C), ubiquitinated forms were detected in Co-IP assays, and protein stabilization was achieved by Lys→Arg substitutions at predicted ubiquitination sites (Table 1; Fig. 4B, D). However, despite the increased protein stability in transient assays, the stable introduction of these mutant constructs into tomato did not lead to a further significant increase in regeneration efficiency compared with the wild-type GRF–GIF constructs (Fig. 5). To our knowledge, these findings provide the first evidence that GRF–GIF proteins are regulated by the ubiquitin–proteasome system and suggest that the relatively low accumulation of SlGRF4–GIF1b may reflect differential proteasome-associated regulation between orthologous chimeras (Fig. 4C).

*AtGRF5–GIF1* transcripts were more stable than *SlGRF4–GIF1b*, consistent with *in silico* RNA secondary structure predictions (Table 2; Fig. 6A, B) and the known ability of *AtGRF5* to evade miR396-mediated silencing (Liu *et al.*, 2009; Kim and Tsukaya, 2015), whereas tomato GRF genes, including *SlGRF4*, remain targets of Sly-miR396a during regeneration (Park *et al.*, 2024). mRNA secondary structure has long been recognized as a determinant of transcript stability and translational efficiency (Wan *et al.*, 2014). In plants, genome-wide analyses have shown that differences in GC content, structural motifs, and RNA-binding protein interactions contribute to variations in mRNA half-life (Narsai *et al.*, 2007; Miller *et al.*, 2011). These results suggest that, in addition to the well-established miR396-mediated cleavage of GRF transcripts, intrinsic sequence features contribute to the differential persistence of GRF–GIF mRNAs (Fig. 6C). Importantly, the lysine-to-arginine substitutions introduced into the GRF–GIF chimeras were designed to target predicted ubiquitination sites and lie outside the conserved miR396 recognition sequences. *AtGRF5* is inherently insensitive to miR396-mediated repression, and the predicted miR396-binding region in *SlGRF4* is located in a distinct portion of the coding sequence that does not overlap with the mutated lysine residues, indicating that the introduced mutations alter ubiquitination potential without affecting miRNA targeting.

Taken together, these results demonstrate that regeneration efficiency is governed not by a single molecular layer but by coordinated regulation at both the RNA and protein levels. We speculate that AtGRF5–GIF1, characterized by higher transcript levels and greater protein accumulation, promotes earlier transcriptional waves associated with shoot regeneration, whereas SlGRF4–GIF1b, which shows lower protein accumulation, sustains delayed but prolonged responses. This interpretation aligns with the view that multilayered regulatory checkpoints collectively fine-tune regeneration capacity (Shyu *et al.*, 2008; Nelson and Millar, 2015).

Beyond the mechanistic findings, this study also provides insights relevant to the practical deployment of GRF–GIF modules in crop biotechnology. GRF–GIF chimeras are increasingly being incorporated into transformation pipelines to overcome regeneration bottlenecks, and our characterization of transcript and protein stability, as well as ubiquitin–proteasome-associated regulation helps explain why certain GRF–GIF combinations perform better across genotypes. In gene-editing workflows, GRF–GIF cassettes can be co-delivered with CRISPR/Cas reagents in a single T-DNA to boost the recovery of edited shoots. After editing, the GRF–GIF module can be removed through Cre/lox-mediated excision or segregated away to obtain transgene-free progeny. These insights reinforce the value of GRF–GIF as a modular regeneration enhancer and provide a mechanistic foundation for its optimized use in future genome engineering applications.

## Conclusion

In this study, GRF–GIF chimeric proteins were shown to enhance shoot regeneration in tomato, including from true leaf explants, a tissue typically associated with low regenerative competence. Regeneration from leaf explants was markedly improved by both AtGRF5–GIF1 and SlGRF4–GIF1b across cultivars, resulting in increased recovery of rooted, antibiotic-resistant transgenic plants. Transcriptomic and biochemical analyses revealed that GRF–GIF activity is shaped by multilayered regulation at both the RNA and protein levels, including regulation associated with the ubiquitin–proteasome system. Together, these findings demonstrate that GRF–GIF chimeras can extend effective regeneration beyond conventional explant systems and provide a mechanistic basis for their optimized use in tomato transformation.

## Supplementary Material

erag187_Supplementary_Data

## Data Availability

The raw RNA-seq data of the MicroTom GRF–GIF transgenic plants have been deposited in the Korea BioData Station (K-BDS; https://kbds.re.kr/) within the Korean Read Archive (KRA) under accession ID KRA2462887.

## Supplementary data

The following supplementary data are available at *JXB* online.

Fig. S1. Phylogenetic relationships and accession information of GRF and GIF family members from *A. thaliana*, *O. sativa*, *Z. mays*, and *S. lycopersicum*, identifying *SlGRF4* and *SlGIF1b* as the closest tomato homologs of *AtGRF5* and *AtGIF1*.

Fig. S2. Multiple sequence alignment of *AtGRF5*, *SlGRF4*, *AtGIF1*, and *SlGIF1b* highlighting conservation of the QLQ, WRC, and SSXT domains required for GRF–GIF interaction and transcriptional regulation.

Fig. S3. Transient *Agrobacterium*-mediated expression of 35S:eGFP in cotyledons of MicroTom, Doterang, and Gwangbok, confirming susceptibility of all cultivars to gene delivery.

Fig. S4. Phenotypic characterization of T_0_ and T_1_ GRF–GIF transgenic tomato plants, showing normal vegetative growth, flowering, seed development, and early seedling establishment.

Fig. S5. Schematic of GRF–GIF transgene constructs and RT–qPCR primer positions.

Fig. S6. Immunoblot analysis showing poor accumulation of GRF–GIF fusion proteins in regenerated stable transgenic tomato tissues despite confirmed transcript expression.

Fig. S7. Original, uncropped immunoblot data for the MG132 treatment experiments presented in Fig. 4.

Fig. S8. Original western blots for GRF–GIF fusion proteins and ubiquitination site mutants. These blots correspond to the quantification data presented in Fig. 3B and D.

Table S1. List of GRF and GIF family members used for phylogenetic analysis, including gene names and accession numbers.

Table S2. Primer sequences used for cloning, RT-PCR, and RT–qPCR analyses.

Table S3. Quantification of shoot regeneration efficiencies for EV, *AtGRF5–GIF1*, and *SlGRF4–GIF1b* lines across tomato cultivars.

Table S4. RT–qPCR expression values of GRF–GIF transgenes at multiple time points during regeneration.

Table S5. RT–qPCR expression profiles of regeneration- and hormone-related genes during GRF–GIF-mediated regeneration.

Table S6. Shared DEGs between AtGRF5–GIF1 and SlGRF4–GIF1b classified into eight expression patterns.

Table S7. Quantitative counts of DEGs across temporal patterns (D0, D14, D28) and their intersection.

Table S8. Shared GO terms between AtGRF5–GIF1 and SlGRF4–GIF1b categorized into eight functional patterns.

Table S9. Predicted ubiquitination sites in GRF–GIF fusion proteins identified by UbPred analysis.

Table S10. Quantification of GRF–GIF protein accumulation following MG132 treatment in transient assays.

Table S11. Regeneration efficiencies of tomato lines expressing ubiquitination site–mutant GRF–GIF constructs.

Table S12. mRNA decay constants and half-life estimates derived from transcriptional shutoff assays.
