# Supplementary material for: Chimeras of GROWTH-REGULATING FACTOR and GRF-INTERACTING FACTOR enhance leaf-based regeneration and transformation efficiency in tomato
Source: J Exp Bot. 2026 Apr 22;77(14):4579–93. doi: 10.1093/jxb/erag187 (PMC13415958; doi:10.1093/jxb/erag187)
Supplement: erag187_Supplementary_Data [file erag187_supplementary_data.zip › jexbot316906-file001.pdf]

Supplementary Figures

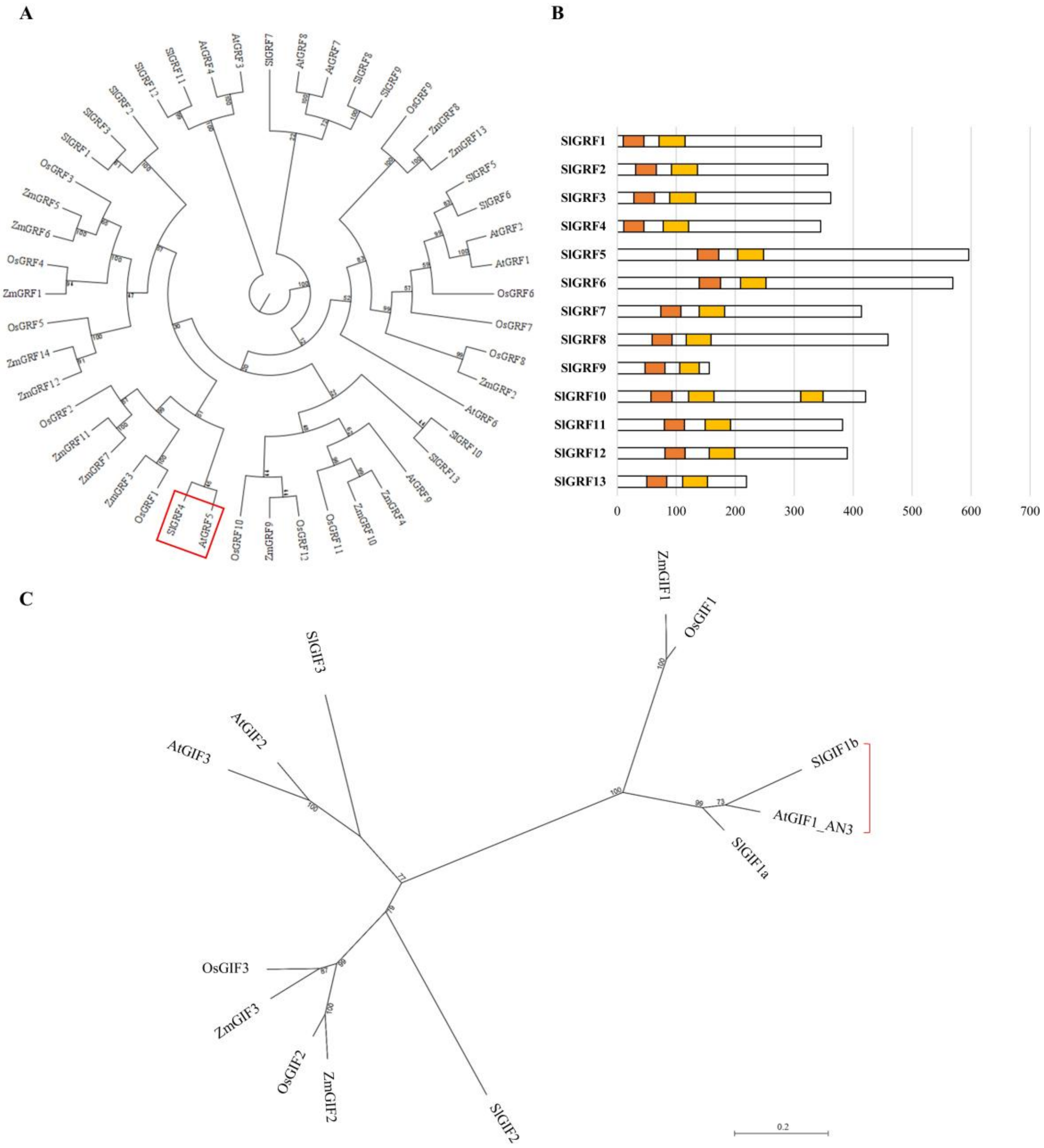

Supplementary Fig. S1. The information of *GRFs* and *GIFs* in tomato. Phylogenetic trees of *GRF*(A) and *GIF*(C) families for tomato, *Arabidopsis*, rice and maize. B. The QLQ (orange) and WRC (yellow) domain of *SlGRFs* in tomato. Tomato (*SlGRFs* and *SlGIFs*), *Arabidopsis* (*AtGRFs* and *AtGIFs*), rice (*OsGRFs* and *OsGIFs*) and maize (*ZmGRFs* and *ZmGIFs*). The phylogenetic tree was established with entire protein sequences from the above plant species by the MEGA11 version the neighbor-joining tree method following the pair-wise deletion method. The numbers on the branches indicate bootstrap support values from 1,000 replications. The protein sequences used in the phylogenetic analysis are listed in Supplementary Table 1 with their accession numbers.

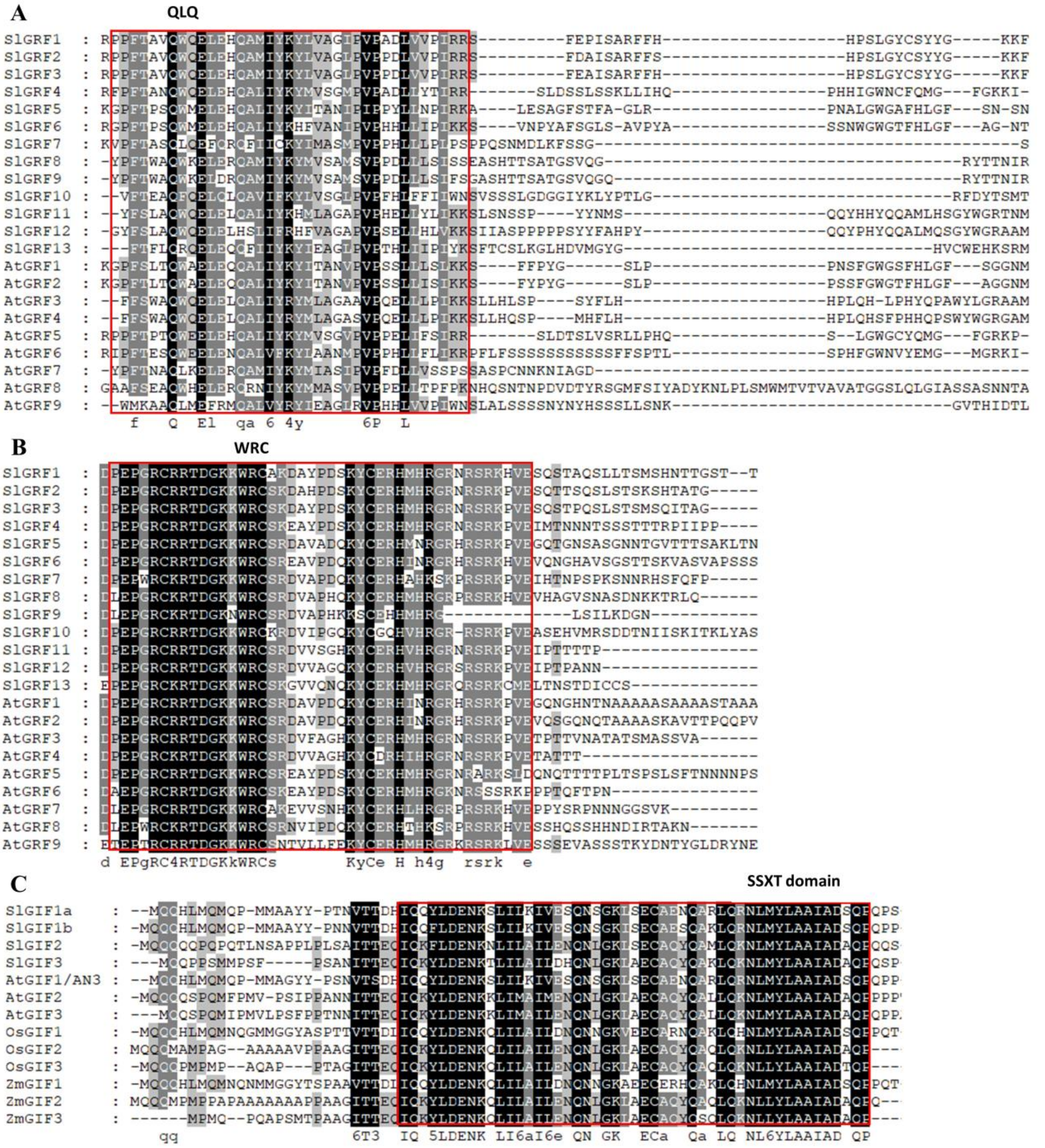

Supplementary Fig. S2. Sequence alignment of GRF, GIF proteins from various species. Tomato (*S. lycopersicum*), Arabidopsis (*A. thaliana*), rice (*O. sativa*) and maize (*Z. mays*). (A) , (B) and (C) the QLQ, WRC and SNH domains (respectively) are in red box. The amino acids multiple alignment by the MEGA11 version ClustalX following the GeneDoc software.

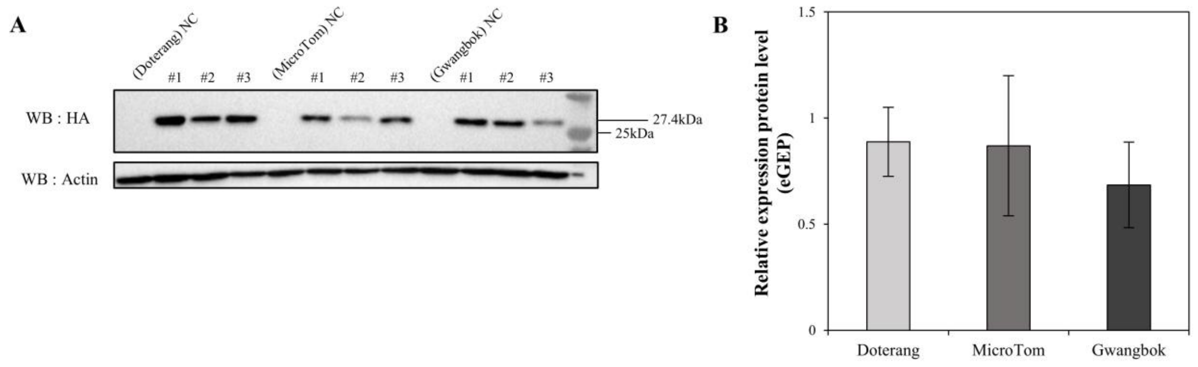

Supplementary Fig. S3. Transient *Agrobacterium*-mediated expression of 35S::eGFP in cotyledons of three tomato cultivars. Cotyledons from Doterang, MicroTom, and Gwangbok seedlings at the same developmental stage were infiltrated with Agrobacterium tumefaciens carrying a 35S::eGFP construct. Tissues were harvested at 3 days post-infiltration (dpi) and total proteins were analyzed by immunoblotting. (A) Representative immunoblots showing eGFP (anti-HA) and Actin (loading control) for three independent 3 biological replicates per cultivar. (B) Densitometric quantification of eGFP signals normalized to Actin and expressed relative to the mean value of MicroTom. Bars indicate mean ± SD (n = 3).

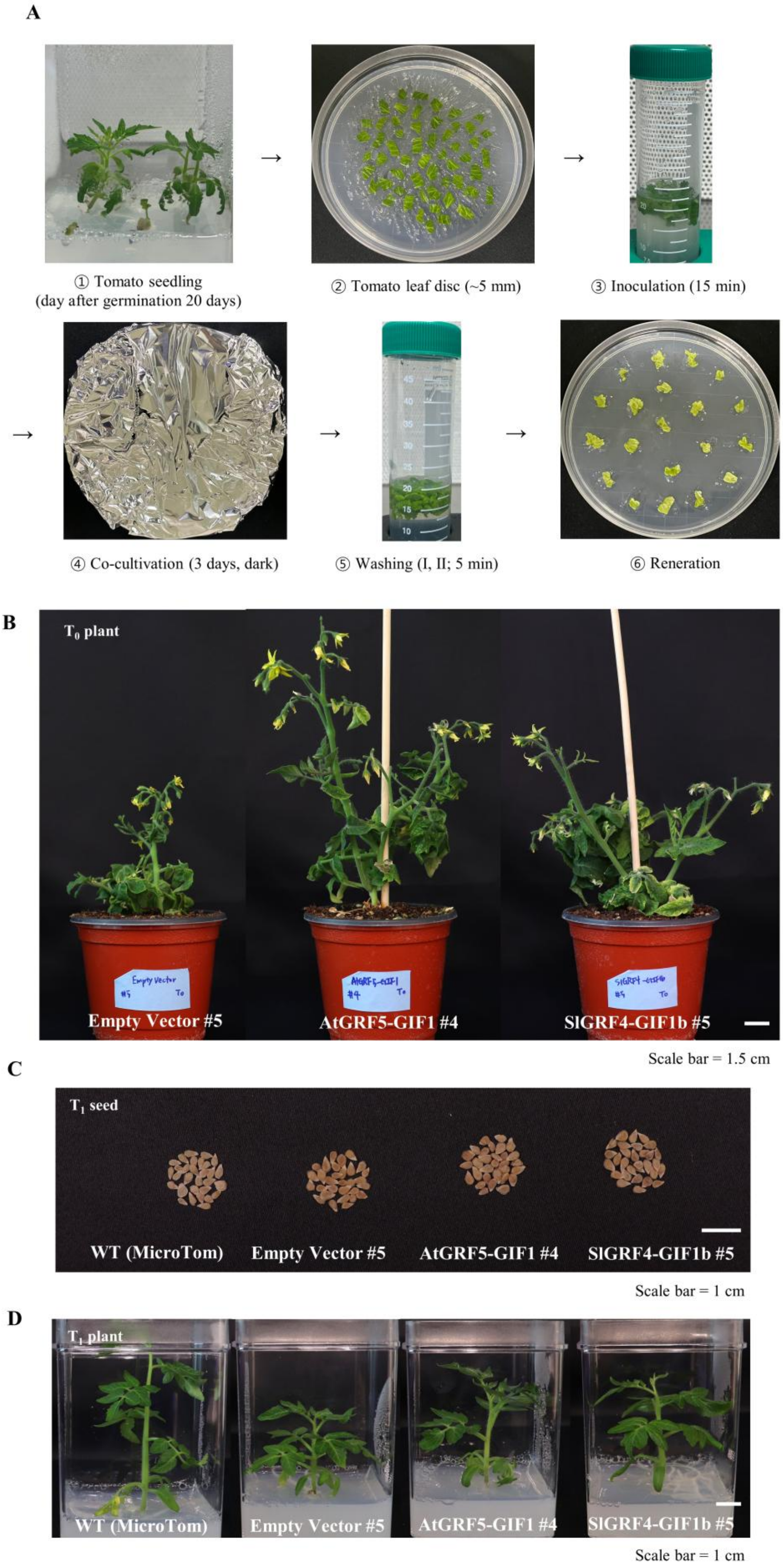
A
① Tomato seedling
(day after germination 20 days)
② Tomato leaf disc (~5 mm)
③ Inoculation (15 min)
④ Co-cultivation (3 days, dark)
⑤ Washing (I, II; 5 min)
⑥ Reneration
B
T_0 plant
Empty Vector #5
AtGRF5-GIF1 #4
SlGRF4-GIF1b #5
Scale bar = 1.5 cm
C
T_1 seed
WT (MicroTom)
Empty Vector #5
AtGRF5-GIF1 #4
SlGRF4-GIF1b #5
Scale bar = 1 cm
D
T_1 plant
WT (MicroTom)
Empty Vector #5
AtGRF5-GIF1 #4
SlGRF4-GIF1b #5
Scale bar = 1 cm

Supplementary Fig. S4. Phenotypic characterization of GRF–GIF transgenic tomato plants. (A) Schematic overview of the *Agrobacterium*-mediated leaf explant transformation procedure used in this study, including explant preparation, co-cultivation, and shoot regeneration steps. (B) Representative images of soil-grown T_0 transgenic plants (approximately 9 weeks after regeneration) carrying the empty vector, AtGRF5–GIF1, or SlGRF4–GIF1b constructs. (C) Representative images of an equal number of T_1 seeds (n = 30 per genotype) harvested from PCR-validated T_0 plants; T_1 progeny were further confirmed by antibiotic selection, with no visible differences in seed size or morphology compared with wild type (MicroTom) and empty vector controls. (D) Representative images of T_1 seedlings grown on in vitro culture medium for approximately 5 weeks, indicating normal early vegetative development across all genotypes. Scale bars are indicated in each panel.

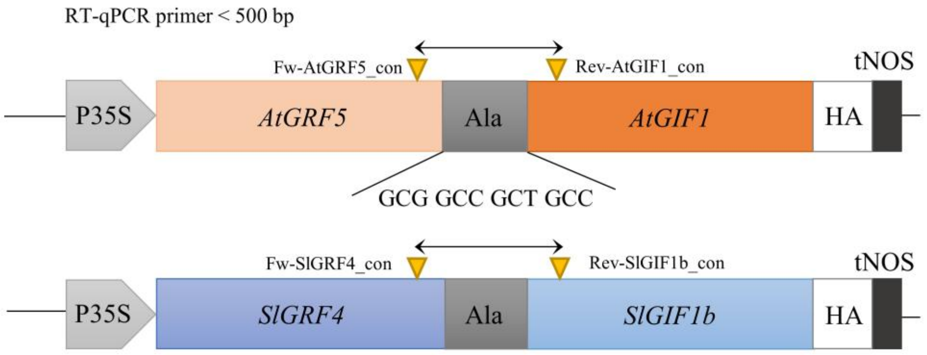

Supplementary Fig. S5. Schematic representation of the GRF–GIF transgene constructs and primer positions used for RT–qPCR. Primers were designed to span the GRF–linker–GIF junction, ensuring specific amplification of the transgene rather than endogenous GRF or GIF loci. Each RT–qPCR sample consisted of a randomized pool of 4–5 explants per biological replicate. Primer amplicons were designed to be < 500 bp.

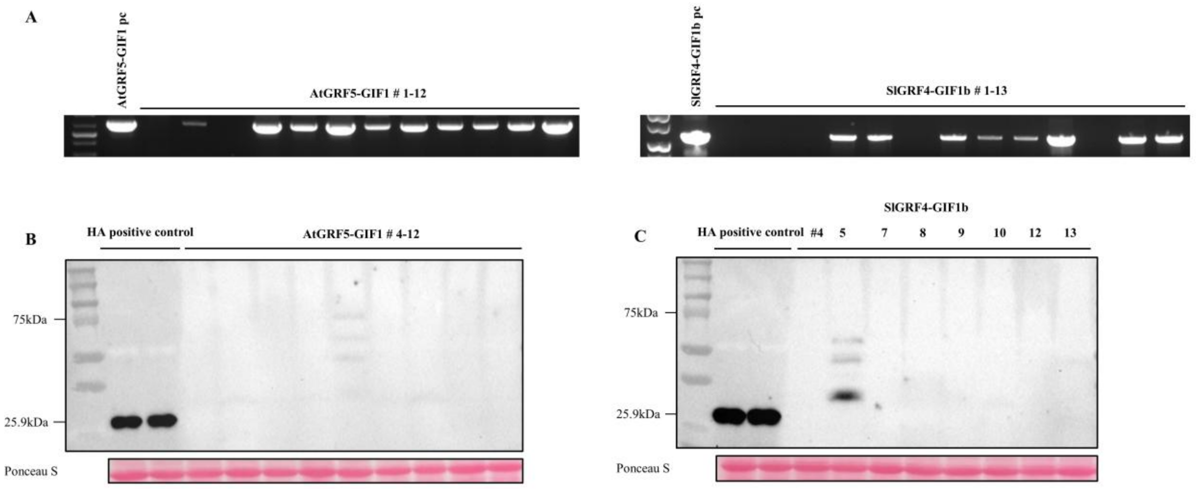

Supplementary Fig. S6. RT-PCR and Western blot analysis of transgenic tomato (T_0) plants. Leaf tissues from regenerated shoots expressing AtGRF5–GIF1 or SlGRF4–GIF1b were used for both RT-PCR and western blot analysis. (A) RT-PCR analysis confirming *AtGRF5–GIF1* and *SlGRF4–GIF1b* transcript accumulation in regenerated shoots. Western blot analysis of AtGRF5–GIF1 (B) and SlGRF4–GIF1b (C) lines using anti-HA antibody. An HA-tagged DsRed protein transiently expressed in *N. benthamiana* was used as the HA positive control. Despite clear mRNA expression, HA-tagged proteins were not detected, indicating rapid protein turnover. Ponceau S staining shows equal protein loading.

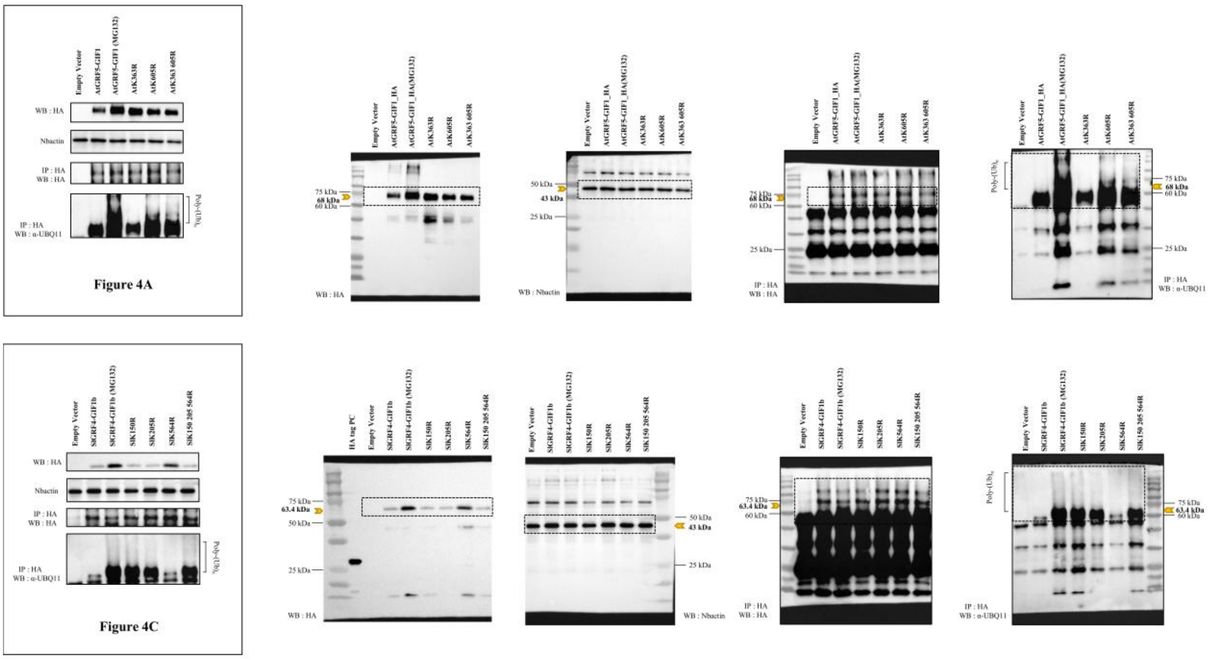

Supplementary Fig. S7. Original western blot images for Figure 4A and 4C. Western blot images showing the original, unprocessed data for the ubiquitination and protein stability assays of GRF–GIF fusion proteins. These include full membrane scans for AtGRF5–GIF1 and SlGRF4–GIF1b, as well as site-directed mutagenesis constructs. Dashed boxes highlight relevant protein bands, and yellow arrows indicate molecular weight markers.

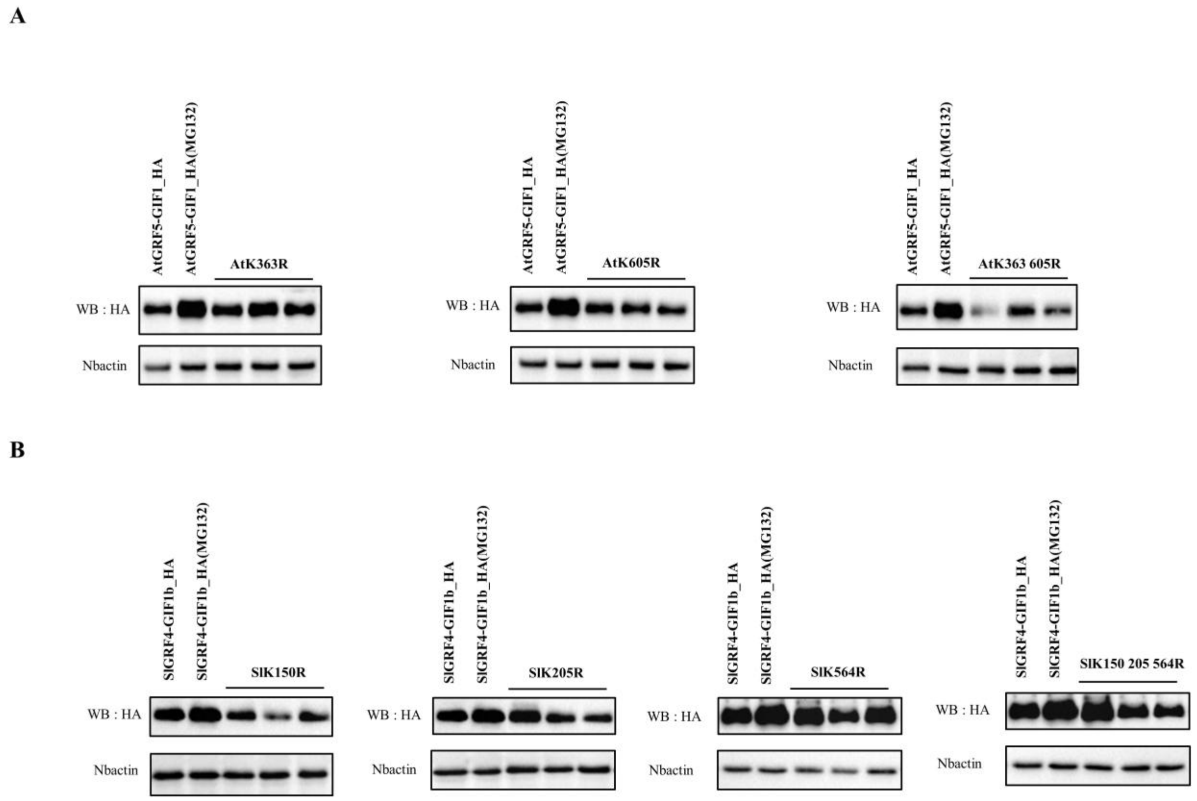

Supplementary Fig. S8. Original western blot images used for quantification in Figure 4B and 4D. These images represent protein expression levels of GRF–GIF fusion constructs and their ubiquitination site mutants. Blots were probed with anti-HA to detect GRF–GIF fusion proteins and with anti-ACTIN as a loading control. Quantification of band intensity was performed using ImageJ.
